# The Venereal Peril

**Published:** 1907-09

**Authors:** H. McHatton

**Affiliations:** Macon, Ga.


### Journal-Record of Medicine

Successor to Atlanta Medical and Surgical Journal, Established 1855, and Southern Medical Record, Established 1870.

OWNED BY THE ATLANTA MEDICAL JOURNAL CO.

Published Monthly.

*Official Organ Fulton County Medical Society, State Examining Board, Presbyterian Hospital, Etc.*

VOL. IX. SEPTEMBER, 1907. No. 6

BERNARD WOLFF, M. D., EDITOR. 319-320 Prudential Bldg.

GEORGE BROWN, M. D., BUSINESS MANAGER. Business Office 1½-5½ S. Broad St.

E. G. BALLENGER, M. D., ASSOCIATE EDITOR AND ASSISTANT BUSINESS MANAGER.

### ORIGINAL COMMUNICATIONS.

### THE VENEREAL PERIL.*

By H. McHatton, M. D., Macon, Ga.

I consider the subject assigned to me to-night, one of extreme importance, one of which you should have full knowledge, so that you will not fall into errors, from which you may suffer for your entire future life. It is incompresensible to me, that, with the educational facilities of our country along all other lines, this subject is absolutely tabooed and you are denied all sources of proper information.

You can get no instruction from your teachers, your church, and as a rule, your parents. The consequence is, that a large percentage of you are ruined through absolute ignorance, and through no fault of your own. Your only means of oral information are through your older companions or associates and servants, or written information through quack advertise-

*An address to the Young Men's Christian Association.

ments, with which the country is flooded, and you especially are supplied, as you are one of the large contributors to the maintenance of the parasites of society.

It has always been my opinion, that instruction in regard to your sexual self should be given you at the proper time by your parents—fathers to sons and mothers to daughters. Unfortunately in many instances your parents are but little better informed than you are, and in other instances will not understand your rights to this knowledge. As soon as my own son was of the proper age, I told him all that he should know along these lines, stating that, should he get into trouble from ignorance, it was my fault, but if, from pure perversity, it certainly was his. His instruction has proved most satisfactory to both of us. In my opinion, there is not enough accord between parents and children in our present type of life. It would be much better for both fathers and sons, if they were on terms of more absolute intimacy and companionship, than is the custom among us.

What I shall tell you to-night will be absolute facts, about which, there can be no question among scientific men, and should I err at all, I can assure you, that it will be upon the side of conservatism.

First, we will take up the natural phenomenon of your sexual selves, to which your attention has been attracted in the ordinary course of nature, and to which, as a rule, you attach most undue importance.

Nocturnal emissions begin with puberty and end with normal decline of sexual power, provided, that normal sexual intercourse is not indulged in, and by normal intercourse, I refer to that of married life only. A married man lives with his wife for years, without having emissions, but should a temporary separation occur, for even a short time, his emissions return to stop again on resumption of the married relation . In the unmarried, who do not have their thoughts more or less constantly fixed on their sexual organs, nocturnal emissions occur twice a week to once a month, depending on the personality of the given individual. They are of absolutely no importance, perfectly natural, and bear no relation to your present or future sexual capacity.

SELF ABUSE.

I can almost say with safety, that all boys have been guilty of this vice, at some period of their lives, but fortunately, they have not become habitual masturbators, as their natural self-respect has, in the vast majority of cases, compelled them to stop before material damage has been done, and the chronic habitual victim of self-abuse, as depicted in most of the literature accessible to you, is a very rare individual. The occasional lapses of the majority leave no lasting damage, but if persisted in this vice can only lead to eventual ruin.

Spermatorrhea or the unconscious loss of semen. The real stronghold of the quack is such a rare disease, that the average doctor never sees a case and even the specialist of large experience, but rarely sees one. You know the mental torture, that most of you have suffered from this disease, and the inevitable future calamity that has been held before you. If, under certain conditions, you have a small amount of moisture, perfectly natural, of the parts, you have it. If after urination, there is a small drop of mucus passed, the natural secretion of the deep glands, you have it again. If after a constipated action from the bowels, another drop of mucuous appears, the natural secretion squeezed out of a healthy gland, you have got it in the worst form. If this is not satisfactory, all you have got to do it to put some of your urine in a clean bottle and put it on the mantle piece for forty-eight hours. If a thin white cloud appears in it, during that time, your condition is horrible. As this cloud will appear in nearly every specimen and is only the natural mucus from the bladder, it need not alarm you at all. For some twenty-five years I have been looking for a case of this disease, and have examined any number of young men whose lives had been rendered a constant torture by believing that they had it, but I still have my first case to see.

Now you can see what a mark your ignorance makes of you for the quack. He knows that you have nocturnal emissions, that you have masturbated, and is in a position to prove to your satisfaction, that you have spermatorrhea. No matter how healthy you are, there are always some sensations or slight irregularity about your bodily functions, which every one has, more or less, that by fixing your mind are so increased that you are a prime candidate for the lunatic asylum, or at least a life of impotency. Hundreds of men have consulted me, who had suffered the mental torments of the damned for years—who in their imagination were incapacitated for business or pleasure, and in their own estimation absolutely debarred from marriage, simply because they had nocturnal emissions, and in their early youth had temporarily lapsed into masturbation. Every normal sensation that they had and every slight ailment they even had, could only be attributed, in their estimation, to these causes. The daily advertisements in the papers, the mailed pamphlets on lost manhood and manhood restored. which are written for their special benefit, with the fore-knowledge of what had happened to them—they having no means of learning the truth, had had the desired effect, they were in a condition of nervous fear that was pitiable. It is probably needless for me to state, that the condition of this class of unfortunates is absolutely dependent on their nervous condition and that any doctor can cure them without medicine, just as soon as he can get their confidence.

### CONTINENCE.

Now we come to another question on which most of you have wrong impressions. Is absolute continence conducive to health? POSTIVELY and ABSOLUTELY YES. Is illicit intercourse in any way or at any time desirable or beneficial? ABSOLUTELY NO. Can the danger of infection during illicit intercourse be prevented, either by the selection of any known medical applications? ABSOLUTELY NO. You take your chances and none of you realize, until too late, how horrible they are. The selection of your partner means nothing. The woman that will cohabit with one man, will usually have the same relations with others. By many authorities, the clandestine prostitute is considered more dangerous than the professional. Paradoxical as it may sound, the professional depends on her reputation for her living, if she is infected, it is soon found out by her clientele, and she is financially ruined. The clandestine obviously has not the same regard for her reputation and does not take the same care of her person. A woman can have all three of the venereal diseases in the most contagious stage at the same time, and be ignorant of the fact. The early use and especially abuse of the sexual power, is more liable to bring future trouble, than many other things more dreaded by you.

The young man who astonishes his mate by the graphic description of his sexual powers—priding himself on attributes in which the jackass is by far his superior—in future years, in normal life, will be far the inferior of his continent friend of the present. All men who train for special feats of physical endurance or strength are required by their trainers to abstain from intercourse. Can you find a more complete illustration of the results of continence, than the physical condition and mental power of the men, members of religious orders in which continence is absolute, and in most cases has been so for the entire life of the given individual.

No, my friends, where not one valid reason can be given in justification of incontinence. I could continue indefinitely giving absolutely unquestionable reasons why you should be continent, entirely from a physical standpoint, leaving out the question of the moral responsibilities, on which you get instruction from other sources. It has always been incomprehensible to me why there should be two codes of morals—one for man and one for woman. A woman should have every reason to expect virtue in a man, that he has to expect it in her, and if she knew one thousandth part of the suffering that is brought upon her sex by the past of her husbands, she would certainly demand it.

### VENEREAL DISEASE.

Venereal diseases are in normal condition the most common of all diseaes. It is a great pity that their prevalence cannot be made public. One of the causes of their prevalence is their concealment. If any other diseases as potent for harm, as contagious, and as prevalent existed in this city, a wail would go up, that would be heard all over the civilized world, and if there were no other way of controlling the situation, this city would be depopulated. More anguish and suffering, both mental and physical, to the guilty and the innocent, are caused by these diseases in this city, as well as all other cities, day in and day out, year in and year out, than by any other group of diseases, I could with almost safety say, that with all others combined. This condition of affairs can only be appreciated by the man who practiced general medicine for years, and has thus become familiar with the full facts as no other man possibly can. It is impossible for him to give to an unprofessional mind even an idea of the horrors that he sees daily dependent on veneral diseases, contracted in many instances before he was born. Could such a condition of affairs exist in connection with any other public menance in this country? Of course not. But in our increasing civlization and decreasing morality, such diseases should be absolutely ignored; you should not be taught anything about them. We should pray that they do not exist, and even bring ourselves to believe that they do not, if we can. They are horrid, vulgar, dirty, no respectable person should ever hear of them. In our civilization and supposed morality, such things cannot exist, and should not be recognized, if they do. This group of diseases are three in number.

### GONORRHEA.

Until a few years ago, when the gonococcus was discovered, supposed to be a purely local disease, now known that it may be general, to cause more incurable lesions than syphilis and to eventually kill more people, and in the long run cause more suffering so prevalent, that some authorities state that 90 per cent.

of the male populations of the larger cities have been infected. A young man, often only a boy, comes into your office and announces with even a certain degree of pride: "Doctor, I've got the clap, do you think you can cure it in a week?" I always look upon these monuments of ignorance with pity and as a rule, cannot help forming a mental picture of the future possibilities of the poor unfortunate. His discharge may be cured in a week, and may last a year under the best of treatment. He is liable to have a stricture, that will begin to prove of some inconvenience, in any where from three to twenty years, which will in many cases demand a serious operation, and in the words of the late Dr. Van Buren, you will dilate his canal to the full extent, teach him to pass a sound himself, and tell him to pass it every Sunday morning for the rest of his life, otherwise, the stricture will return. He may get the gonococcus in the general circulation and suffer for years from a gonorrheal rheumatism from which he can get no relief. He may have a swelling of one or both testicles, which for a few weeks will give him a definite idea of the amount of suffering a human being can stand and not die. This is usually followed, when both are infected, by permanent and incurable sterility. This being the most common cause of sterility in man.

He may get an infection of the eyes and lose one or both in a few hours. These are only a few of his possibilities, even under the care of the best specialist in the world. We will grant that he is fortunate and escapes by an especial dispensation of Providence all these possibilities. His discharge disappears, he thinks he is well, and has no reason to think otherwise. His wilds oats are a thing of the distant past; he has reformed, and is one of the rising young men of his class: he falls in love; becomes deeply infatuated; has found the ONLY woman; would sacrifice everthing in the world for her; would inhabit any of the various departments of Hades indefinitely, rather than see her suffer mentally or physically for one instant; he marries, and for the first time in his life begins to have an idea of what the world has to offer in the shape of happiness; his wife becomes pregnant shortly after marriage, his possibilities begin again. In due time she is delivered of the finest baby in the world (if you will take the opinion of the grand parents) a composite picture of the notables of the family on both sides for many generations. Professionally, a good, strong, healthy child. The rejoicing is great, happiness is complete. In a few hours the baby's eyes become inflamed, in a few more the inflammation has become violent, in a few days complete and permanent blindness has taken place, due to the infection of the child's eyes by the gonoccocci in the maternal passages. This is a cause of a larger per cent. of blindness than any other one thing, as it is shown by the statistics of the blind asylums all over the world.

Again, instead of becoming pregnant, his wife contracts a sub-acute gonorrhea, and after months or years of invalidism, she is carried to the operating room and her tubes and ovaries removed, which procedure renders her incable of ever bearing children, and in fact unsexes her. Eminent specialists in these lines state that sixty per cent. of all this class of operations are due to gonorrheal infection. I shall not prolong this list of possibilities, but not for lack of material.

Now what do you think of the man that classes a gonorrhea on a par with a cold and equally harmless,

### CHANCROID.

A chancroid is a local foul ulcer, that often ends in sloughing and considerable destruction of tissue, leaving unsightly permanent scars, of no future importance, but often producing serious illness, and great suffering to the patient.

### SYPHILIS.

For obvious reasons it is impossible to even appreciate the prevalence of this disease. During an examination of prostitutes some years since in New York, two-fifth of them acknowledged voluntarily to be syphilitics. I suppose I have not less than fifty syphilitics under observation at any time, and there are over fifty doctors in this city. You can with safety put the syphilitics of this city down at 2,500. In my opinion 5,000 would come much nearer the mark. If a man contracts this disease, he has at least one certainty, no competent physician will allow him to marry in less than two years, and this period is very liable to be increased to three four, five or even six years, during which time he has to be constantly under observation and treatment. This disease affects every organ and tissue of the body, in fact can be classed as a chronic general infection; is inherited for generations; is one of the main causes of abortion and infant mortality. No one can tell when the active contagion will cease in the given individual. Fournier cites a case where a wife contracted it from her husband thirty-nine years after his inital lesion. Cases of twenty and thirty years are common. According to the latest statistics, twenty per cent. of the woman who have this disease contract it among the highest as well as the lowest.

### INNOCENT VICTIMS.

All sufferers from venereal diseases are liable to infect their associates from a non-sexual standpoint. This danger is much greater from the syphilitics, and they are constantly a menace to the public at large. I have had considerable personal experience with leprosy, and in my opinion the syphilitic is more to be dreaded as a source of danger in the ordinary family relations, than the leper. Many of my professional brethren will differ from this opinion, but this is not the place to argue the point. I have seen an innocent victim of gonorrheal infection lose both eyes from the use of an infected towel. Chancres of the lip are so common that they scarcely cause remark. Those of the tongue, tonsils and finger are no rarity, in fact, they have been noted on nearly all parts of the human body. I have under observation at present four syphilitics, who contracted their disease in a non-sexual manner. A young man may take the stand, that having assumed no responsibilities he has the right to run any risk he sees fit to in promiscuous intercourse. Leaving aside all questions of decency, morality, religion and self respect, from purely the physical aspect, this right I deny en toto.

If the result concerned himself alone or could be confined to himself, it might be a subject of argument. Can he arrogate to himself the right to deny his future wife the blessing of maternity, to compel her to bring into the world a series of dead or degenerate children, to rob her of health, to expose her to the loss of her organs of generation, or to spend the best days of her life nursing a physical wreck in the shape of a husband, or has he the right to expose his mother or his sister to the possibility only of contracting the most horrible of diseases? This is not overdrawn. Physicians see these conditions in some of their numerous aspects daily. Let him expose himself to smallpox in any civilized land in the world, and see what right the law of the land would allow him to make others suffer from possible chances of his exposure.

To recapitulate:

1st. Nocturnal emissions are a normal phenomenon and but rarely of any importance.

2nd. The evils of the results of self-abuse are much exaggerated.

3rd. Spermatorrhea is such a rare condition that it can safely be ignored.

4th. Continence is perfectly feasible and sexual indulgence is in no sense a necessity.

5th. Illicit intercourse cannot be indulged in without the serious risk of venereal infection.

6th. Venereal infection is the most common and by far the most serious in its present and future results than any other danger to which the vast majority of young men are exposed.

If you are virtuous, stay so.

If not, and not infected, STOP.

If infected, put your self immediately in the hands of your family physician. It may take him years to put you in the condition where you can with physicial safety to your future wife and children enter into the state of matrimony.

